# Haemodynamic Effects of Pimobendan during General Anaesthesia in Healthy Senior Dogs: A Prospective, Randomised, Triple-Blinded, Placebo-Controlled Clinical Study

**DOI:** 10.3390/ani13132110

**Published:** 2023-06-26

**Authors:** Ignacio Sández, José I. Redondo, Pablo A. Donati, José Gómez

**Affiliations:** 1 Department of Anesthesiology and Pain Management, Hospital Veterinario AniCura-Vetsia, Leganés, 28914 Madrid, Spain; 2 Departamento de Medicina y Cirugía Animal, Facultad de Veterinaria, Universidad Cardenal Herrera-CEU, CEU Universities, 46115 Valencia, Spain; nacho@uchceu.es; 3 Department of Anaesthesiology and Pain Management, Facultad de Ciencias Veterinarias, Universidad de Buenos Aires, Buenos Aires 1427, Argentina; padonati@yahoo.com.ar; 4 AniCura Iberia Medical Department, 28006 Madrid, Spain; jose.gomez@anicura.es

**Keywords:** pimobendan, anaesthesia, cardiac output, haemodynamic, oesophageal Doppler

## Abstract

**Simple Summary:**

Pimobendan is a drug that improves cardiac output and contractility in dogs with mitral valve disease and dilated cardiomyopathy, as well as in healthy adult dogs. The intravenous injectable formulation has shown immediate cardiovascular effects in dogs without pro-arrhythmogenic effects. Our study aims to assess the haemodynamic effects of the intravenous administration of pimobendan at a dose of 0.15 mg/kg intravenously in healthy senior dogs undergoing general anaesthesia for surgical procedures. The results of this study showed that pimobendan improves cardiac function parameters measured with an oesophageal Doppler monitor in senior dogs with no previous heart disease. Pimobendan could be used during general anaesthesia in senior dogs when there is concern about the possible presence of impaired cardiac function. There were no significant changes in blood pressure or heart rate. In the present study, continuous haemodynamic monitoring showed that the effects appeared from the first minute and continued for at least 20 min.

**Abstract:**

Pimobendan is an inotropic and vasodilator drug with no sympathomimetic effects. This study aimed to evaluate the haemodynamic effects of pimobendan during anaesthesia in healthy senior dogs. A prospective, randomised, triple-blinded, placebo-controlled clinical study was conducted. Thirty-three dogs (median [range]: 9 [7, 12] years) were anaesthetised for surgical procedures. The dogs were randomly allocated into two groups: eighteen dogs received intravenous pimobendan at a dose of 0.15 mg/kg (PIMOBENDAN), and fifteen dogs received intravenous saline solutions at a dose of 0.2 mL/kg (PLACEBO). Data were recorded before, 1 min, 10 min, and 20 min after injection. Velocity-time integral (VTI), peak-velocity (PV), and mean-acceleration (MA) were measured using an oesophageal Doppler monitor (ODM). Heart rate and mean arterial pressure were also registered. The data were analysed using a two-way ANOVA for trimmed means. Statistical differences were considered if p < 0.05. Twenty minutes after injection, the VTI (13.0 cm [10.4, 22.3]), PV (95.0 [83.0, 160] m/s), and MA (12.6 [9.40, 17.0] m/s^2^) were significantly higher in the PIMOBENDAN group compared to the PLACEBO group (VTI: 10.5 [6.50, 17.4] cm, PV: 80.0 [62.0, 103] m/s and MA: 10.2 [7.00, 16.0] ms^2^). No significant differences were observed in the rest of the variables. Using pimobendan during anaesthesia increases VTI, PV, and MA, as measured by an ODM.

## 1. Introduction

Advances in medicine, surgery, and diagnostic imaging have increased the number of dogs requiring general anaesthesia. Although exact figures are unavailable, the number of elderly patients presenting for anaesthesia appears to be increasing [1,2]. Anaesthesia is a state of controlled nervous system intoxication, which can be life-threatening to even healthy animals [3]. Anaesthetic morbidity in healthy senior patients increases due to a decline in organ reserve. Under surgery and, hence, stress, the diminished reserve capacity of elderly patients impairs their response to increased demand [2]. Moreover, virtually all anaesthetic drugs cause hemodynamic disturbances. A persistent complication associated with the pharmacodynamic properties of anaesthetics is a drop in cardiac output (CO), leading to decreased blood pressure and reduced tissue perfusion, with potentially fatal consequences [3,4,5]. In addition, during severe inflammatory and infectious diseases, which often lead senior dogs to the operating room, the myocardial contractile function may be reduced as well [6].

Inotropic and vasopressor drugs, such as dobutamine, dopamine, or noradrenaline, are commonly used to improve CO and thus correct these imbalances. However, the administration of these drugs has some potential risks. Cardiac arrhythmias and severe blood pressure fluctuations are expected when used [7,8]. As these catecholamines have a short half-life, they must be administered through continuous infusion using pumps or syringe pumps. This process adds to the complexity and cost of anaesthetic [4,7,8,9].

Pimobendan is a drug with a dual effect: it produces sensitisation of intracellular calcium (Ca^2+^), which has a positive inotropic effect, while also inhibiting phosphodiesterase III (PDE III) activity, thus creating a relaxing effect on cardiac and peripheral vascular musculature [10,11]. These effects on the cardiovascular system have made this drug a mainstay in treating the preclinical and clinical phases of mitral valve disease and dilated cardiomyopathy in dogs and reducing morbidity in animals with chronic heart disease [11,12,13,14]. An intravenous injectable formulation has recently been registered. Laboratory studies on this formulation have shown immediate cardiovascular effects in dogs without pro-arrhythmogenic effects, making this drug a valuable tool in managing acute congestive heart failure [15].

Previous studies have shown that the increase in cardiac contractility from pimobendan is similar to that provided by dobutamine but with a longer duration of effect. This fact, combined with the absence of pro-arrhythmogenic effects, makes pimobendan a beneficial drug for improving CO and achieving hemodynamic stabilisation in a sustained manner over time [16,17,18,19].

Invasive and non-invasive methods used to study the hemodynamic effects of inotropic drugs include the pulmonary artery catheter, thermodilution, intraventricular pressure catheter, and echocardiography [10,11,16,17,18,19]. While invasive techniques are still the gold standard for hemodynamic assessment, they are also associated with high morbidity [20]. Non-invasive and minimally invasive techniques are frequently used in clinical settings for this reason. The oesophageal Doppler monitor (ODM) is a minimally invasive technique which has been used in different clinical settings [20,21,22,23] and has good agreement with transthoracic echocardiography [24].

To the best of the authors’ knowledge, to date, there have been no clinical studies evaluating the effects of intravenous pimobendan in healthy anaesthetised senior dogs during surgical procedures.

The aim of the present study was to assess the specific effects of intravenous administration of pimobendan on left ventricular function in healthy senior dogs undergoing general anaesthesia for surgical procedures. We hypothesise that pimobendan could improve the hemodynamic profile of animals under general anaesthesia by increasing systolic output and cardiac contractility.

## 2. Materials and Methods

### 2.1. Study Design

A prospective, randomised, and triple-blind clinical trial was conducted. This study was approved by the Animal Experimentation Ethics Committee of CEU Cardenal Herrera University in Valencia, Spain (CEEA19/23). The owners of the animals used in the study were informed about their objectives and risks and signed an informed consent form. The animals were treated by the ethical principles set out in the Animal Welfare Act [25].

### 2.2. Inclusion/Exclusion Criteria

Dogs seven years of age or older, of any breed and body condition, weighing between 5 and 25 kg and undergoing a surgical procedure were included. To determine the patient’s health status and ASA classification, a pre-anaesthetic assessment was carried out 24 h before the procedure, including medical history, physical examination, blood analysis (haematology and biochemistry), two chest X-rays, and an electrocardiogram.

Dogs were excluded from the study if the assessment showed abnormalities in the medical history or physical examination or if their ASA classification was III or higher. Animals with tachycardia (HR > 180 bpm), bradycardia (HR < 60 bpm), arrhythmias, or a heart murmur detected on auscultation were also excluded. Lastly, animals with pre-existing oesophageal malformations and those with diseases at risk of oesophageal bleeding, such tumours, were excluded from the study.

### 2.3. Triple-Blind Study Design

To ensure randomisation and prevent study bias, a system was set up in which four independent steps were performed without any communication between the four authors concerning their functions.

One of the investigators (JG) prepared samples by assigning random codes to vials containing either the placebo or pimobendan and labelling the vials with the codes. A randomisation sequence by permuted blocks of 10 with an allocation of 1:1 was generated by a web page (www.random.org). A safety checklist with these codes was drawn up to identify the substance administered in the event of a complication during the study. All the vials were mixed in a single container and sent to another investigator (IS) who performed all the anaesthetic procedures. The person who performed the anaesthetic procedures, sample administration and data collection needed to learn the coding system. This individual only identified the product code administered to each patient and collected the corresponding data. As no adverse events occurred during this phase, the safety checklist did not need to be used.

After the number of cases required for statistical analysis had been reached, the investigator performing the anaesthetic procedures (IS) sent case numbers and codes of the vials administered, but not the data to be assessed, to another investigator (JG). The data recorded during the anaesthetic procedures were sent to a third investigator (JIR) for statistical analysis, with groups identified as A and B.

After the data from groups A and B were analysed and the statistical results were obtained, the treatment assignments were unblinded to determine to which study group (pimobendan or placebo) each case belonged.

### 2.4. Anaesthetic Protocol

The animals were kept on a 12 h solid and liquid fast before anaesthesia.

Premedication consisting of 3 µg/kg of dexmedetomidine (Dexdomitor, Ecuphar, Barcelona, Spain) and 0.3 mg/kg of methadone (Semfortan, Ecuphar, Barcelona, Spain) was administered via the intramuscular route. After 20 min, the forelimb was shaved to place a catheter in the cephalic vein. The same was performed in the tarsal area to place a catheter in the dorsal pedal artery. A 3 mL kg/h of Ringer’s lactate intravenous (IV) solution (Lactato RingerVet, BBraun Vetcare, Barcelona, Spain) was administered throughout the procedure.

Pre-oxygenation was administered for 5 min with 100% oxygen (3 L/min) via face mask, after which alfaxalone (Alfaxan, Dechra Veterinary Products, Barcelona, Spain) was administered in aliquots of 0.5 mg/kg every 30 s until loss of the palpebral reflex was achieved. Orotracheal intubation was then performed. Isoflurane (IsoFlo, Ecuphar, Barcelona, Spain) was administered in 100% oxygen (1 L/min) using a semi-closed circle circuit. The concentration of isoflurane was adjusted based on assessing the depth of anaesthesia (eyeball position, presence of palpebral reflex, and jaw tone). All animals were mechanically ventilated using volume-controlled ventilation (Drager Cicero, Lübeck, Germany) with a tidal volume of 15 mL/kg, a positive end-expiratory pressure (PEEP) of 4 cmH_2_O, an inspiratory pause of 30%, and a respiratory rate that was sufficient to maintain normocapnia (end-tidal carbon dioxide tension of 35–45 mmHg).

During the stabilisation of anaesthesia, the regional anaesthetic block needed for surgery was applied.

Animals were placed in either dorsal or sternal recumbency, depending on the type of surgical procedure.

### 2.5. Monitoring

A multiparameter monitor (Drager Vitara 8060 PM, Lübeck, Germany) was used to continuously monitor heart rate (HR), blood-oxygen saturation (SpO_2_), end-tidal carbon dioxide tension (PE’CO_2_), end-tidal of isoflurane (FeISO) and rectal temperature, which were recorded every minute. Systolic, diastolic, and mean arterial pressure (MAP) were monitored invasively using an arterial pressure transducer, previously calibrated at the animal’s phlebostatic axis and recorded every minute.

The 4 MHz, 3 mm probe (MP50, Deltex Medical, Chichester, UK) of an oesophageal Doppler monitor (ODM, CardioQ, Deltex Medical, Chichester, West Sussex, UK) was then introduced caudally in the mouth and advanced until its tip was judged to be located at the distal third of the oesophagus, using the reference marks appearing on the probe. The quality and sound of the Doppler signal were used as guides to determine the optimal location for placement of the oesophageal probe, where the descending aorta runs parallel to the oesophagus and, thus, where the maximum aortic flow velocity is detected [20,21,22,23,24]. Left ventricular function was then assessed by monitoring peak velocity (VP, maximum blood velocity in the aorta), velocity-time integral (VTI, distance blood travels per beat), minute distance (MD, distance blood travels per minute), and mean acceleration (MA, mean acceleration of blood in the aorta during ejection). The recorded data were the average of 10 beats on the ODM.

### 2.6. Data Collection

Animals were randomly assigned beforehand to two groups called PLACEBO and PIMOBENDAN, depending on the drug to be administered during the study.

After adequate haemodynamic stability was achieved, defined as HR and BP being within normal ranges (HR > 50 and < 120 bpm, and MAP > 60 and < 100 mmHg), and without fluctuations exceeding 25% for at least 5 min, and at least 10 min after induction, data collection began.

All parameters were recorded during surgical procedures at four different times: 1 min before drug or placebo administration (T0), 1 min after drug or placebo administration (T1), then 10 (T2) and 20 min after administration (T3).

Patients in the PIMOBENDAN group received a dose of 0.15 mg/kg of pimobendan via IV, and animals in the PLACEBO group received a dose of 0.2 mL/kg of saline solution (in the same total volume as was administered to the PIMOBENDAN group) for one minute. All cases in the study were performed by the same anaesthesiologist (IS).

### 2.7. Statistical Analysis

The statistical study used R statistical software version 4.3.0 [26]. Cardiac output was taken as the critical variable. A precision of 0.5 L/min (minimum value of the difference to be detected), a variance of 0.3, an alpha error of 0.05, and a beta error of 0.2 were established. Considering these values, the required sample size was 15 animals per group (n = 30).

The effect size was calculated using the pwr.t2n.test function from the pwr-package [27] for groups of exact sample sizes (n1 = 15; n2 = 15). The significance level was set at 0.05; the power was 0.8. An effect size of -0.93 was obtained.

The normality of the variables was verified with a Shapiro–Wilk test. The equality of variances was studied using the Levene test. Neither VTI nor VP or MA met normality and homoscedasticity criteria. Consequently, a robust statistical approach was chosen for comparing groups [28].

Comparison between groups of the studied variables (HR, MAP, VTI, VP, MA, PE’CO_2_, and FeISO) over time (T0, T1, T2, and T3) was performed using the t2way function for independent samples, which computes a two-way ANOVA for trimmed means with interaction effects. Related post hoc tests are in the mcp2atm function [28]. The trim level for the means was 0.2. Data are presented numerically as medians (minimum to maximum), 95% confidence intervals, and graphically as medians, interquartile ranges, and minimums and maximums.

## 3. Results

### 3.1. Demographic Data

A total of 33 animals were included in the study (18 in the PIMOBENDAN group and 15 in the PLACEBO group). Surgical procedures performed were: ovariohysterectomy (n = 10; 30.3%), dental procedure (n = 4; 12.1%), mastectomy (n = 4; 12.1%), anterior cruciate ligament (n = 3; 9%), cystotomy (n = 3; 9%), orchiectomy (n = 2; 6%), perineal hernia (n = 2; 6%), mastocytoma (n = 1; 3%), submandibular gland excision (n = 1; 3%), abdominal cryptorchidism (n = 1; 3%), and palpebral tumour (n = 1; 3%). All dogs were in dorsal recumbency throughout the study, except for two animals in the PLACEBO group and two in the PIMOBENDAN group, which were in sternal recumbency. Demographic data are described in Table 1.

### 3.2. Haemodynamics Parameters

**Figure 1:**
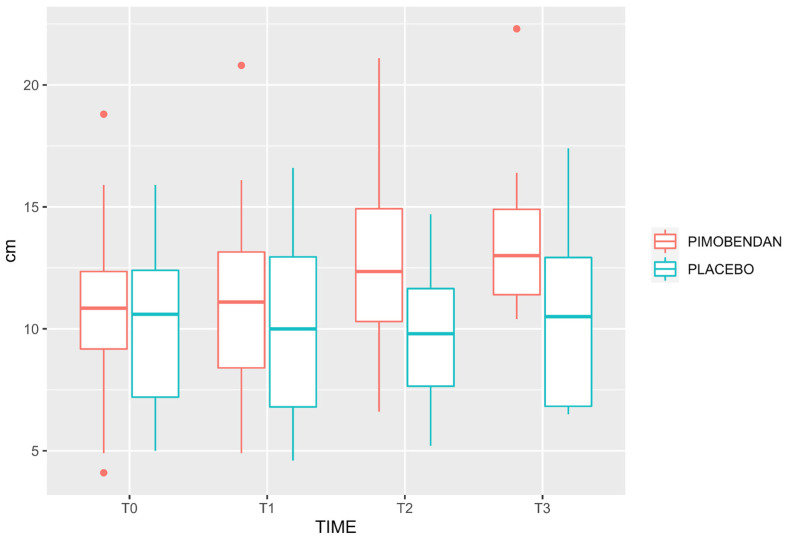
Box and whisker plot for velocity-time integral in PLACEBO and PIMOBENDAN groups over time. The rectangular box represents the interquartile range (IQR), spanning from the first quartile (Q1) to the third quartile (Q3), with a line inside indicating the median. The whiskers extend from the box’s edges and denote the data’s variability. Any data points outside this range are considered outliers and are depicted as individual dots.

**Figure 2:**
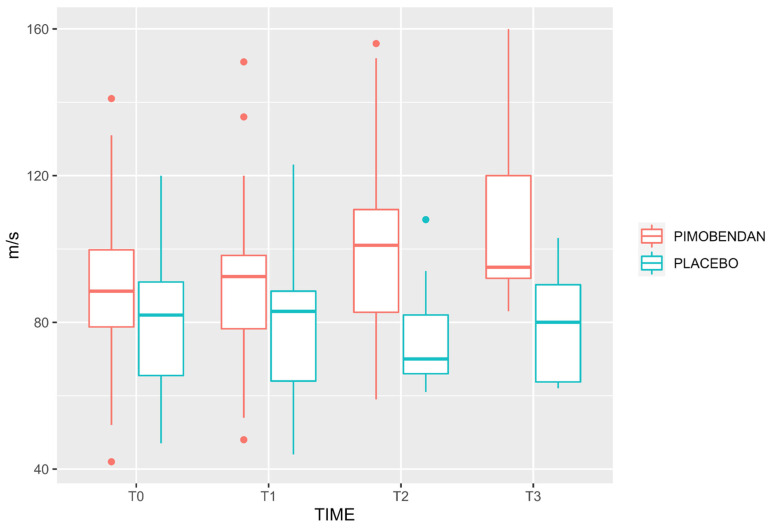
Box and whisker plot for peak velocity in PLACEBO and PIMOBENDAN groups over time. The rectangular box represents the interquartile range (IQR), spanning from the first quartile (Q1) to the third quartile (Q3), with a line inside indicating the median. The whiskers extend from the box’s edges and denote the data’s variability. Any data points outside this range are considered outliers and are depicted as individual dots.

**Figure 3:**
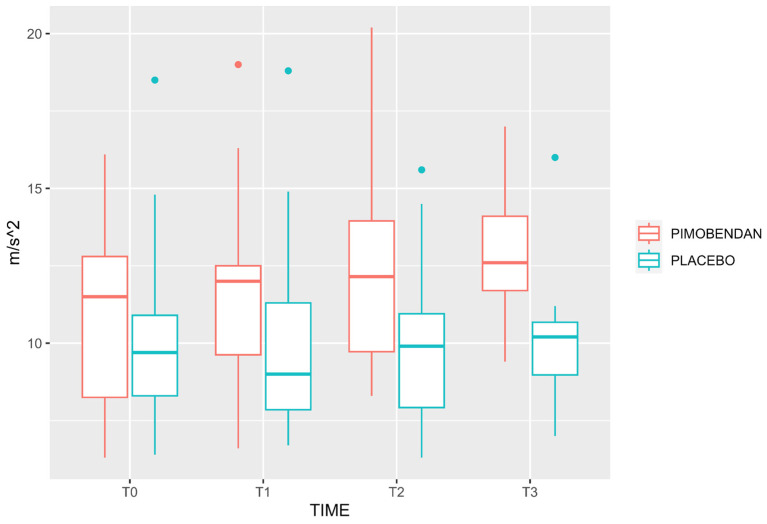
Box and whisker plot for mean acceleration in PLACEBO and PIMOBENDAN groups over time. The rectangular box represents the interquartile range (IQR), spanning from the first quartile (Q1) to the third quartile (Q3), with a line inside indicating the median. The whiskers extend from the box’s edges and denote the data’s variability. Any data points outside this range are considered outliers and are depicted as individual dots.

Velocity-time integral, PV, and MA were higher in the PIMOBENDAN group than PLACEBO group (*p* = 0.019; CI 95%: 1.3–13.9; *p* < 0.0001, CI 95%: 35–95; *p* < 0.0001, CI 95%: 4.8–13.8, respectively). Minute distance showed no difference comparing both groups (*p* = 0.065, CI 95%: −32–1071). Neither were there any differences in HR (*p* = 0.12, CI 95%: −6.9–58), MAP (*p* = 0.19, CI 95%: −53–11), PE’CO_2_ (*p* = 0.23, CI 95%: −1.2–4.7), and FeISO (*p* = 0.08, CI 95%: −0.47–0.28) between groups (Table 2). Figure 1, Figure 2 and Figure 3 show the median, interquartile range, and outliers of VTI, PV, and MA, respectively.

### 3.3. Adverse Reactions

As there were no adverse events, using the safety checklist was unnecessary and triple-blind status could be maintained until the end of the study.

## 4. Discussion

The results of this study show that pimobendan improves cardiac function parameters in senior dogs with no previous heart disease when administered during anaesthesia at a dose of 0.15 mg/kg IV. These results may be clinically relevant. Pimobendan could be used during general anaesthesia in healthy senior dogs when there is concern about the possible presence of impaired cardiac function, as it produces an almost immediate improvement in contractility and systolic ejection flow with a single bolus administration. Consequently, it could potentially reduce the need for continuous infusion of sympathomimetic drugs in certain clinical situations. However, the hemodynamic changes produced by pimobendan were only evident in the parameters measured by oesophageal Doppler and not in BP or HR. In previously published studies, the hemodynamic effects of pimobendan were seen within five to 10 min of administration [15,16]. In the present study, continuous hemodynamic monitoring showed that the effects appeared from the first minute. This rapid onset of hemodynamic effects may be explained by the fact that the active metabolite of pimobendan (ODMP) reaches high plasma concentrations within two minutes of its administration [16]. Moreover, pimobendan may offer an advantage over other inotropic drugs during anaesthesia. Its effects are immediate upon administering a single bolus and continue for at least 20 min without requiring pumps or syringe pumps [15,16,17,18,19].

Pimobendan is an inotropic and vasodilator drug with no sympathomimetic effects [10]. When administered orally, it can improve the hemodynamic status and physical activity in dogs and reduce morbidity in animals with chronic heart disease [11,13]. It has also been shown to increase CO and reduce systemic vascular resistance in dogs with mitral valve degeneration [12]. Nonetheless, its effects during the administration of anaesthesia in dogs without heart disease have only been studied in experimental young and adult dogs using invasive methods [15,16,17,18,19] or echocardiography [29]. Until now, no clinical studies have assessed the effects of pimobendan in healthy senior dogs under general anaesthesia. Furthermore, it is the first time that the use of oesophageal Doppler to assess the hemodynamic changes produced by pimobendan has been described. This monitor consists of a three mm-thick probe inserted into the oesophagus and left in place for long periods for continuous hemodynamic assessment. It has been used to measure CO during anaesthesia in humans, pigs, and dogs [20,30,31]. Furthermore, it has been used to assess left ventricular function [21] and the hemodynamic effects of different drugs and anaesthetic techniques in dogs [23]. However, the CO measured with the ODM disagrees with the thermodilution [20].

For this reason, the CO measurement of the ODM was not used in the present study to assess the hemodynamic state, but rather the parameters derived from the systolic Doppler flow. The effects of pimobendan on inotropy and changes in left ventricular output were evaluated with the descending aorta Doppler parameters provided by ODM: VTI, VP, and MA. A recently published study evaluates the agreement between ODM measurement and transthoracic echocardiography, demonstrating that ODM can be used during general anaesthesia in dogs similarly to transthoracic echocardiography to assess PV and VTI [24]. The ODM is advantageous during general anaesthesia because the probe can be kept in the oesophagus throughout the procedure to monitor aortic flow variables continuously. Moreover, the clinical nature of this study gave this technique an additional advantage since it was not necessary to interrupt and lengthen surgical procedures to assess these hemodynamic parameters.

Velocity-time integral is the distance blood travels in the aorta during systole. This VTI has been used as a surrogate parameter for stroke volume in humans and dogs [32,33,34]. It is also commonly used to determine stroke volume via echocardiography by multiplying VTI by the cross-sectional area of the aorta [35]. This echocardiographic assessment has been used in several studies to assess the effects of pimobendan in dogs [18,29,36]. Minute distance (MD: VTI multiplied by HR) is directly related to CO and has often been used as an alternative method to assess CO [32].

Furthermore, VTI and MD have been used to determine the hemodynamic effects of propofol during the induction of anaesthesia in dogs. It is a valuable tool for assessing changes that occur quickly and minimally invasively [34]. Pimobendan administration caused a significant increase in VTI, indicating an increase in systolic flow and stroke volume. These results are consistent with previous findings in other studies in which echocardiography measured VTI following pimobendan administration in dogs and cats [37,38]. In the present study, MD increased by 25.15% at 20 min. While this increase may have some clinical significance, it was not statistically significant. These results are consistent with other studies [15,37], which also found no increase in CO in anaesthetised dogs after 60 min. However, other studies in anesthetised dogs did show an increase in CO [10,11,12]. Several reasons could explain why no differences were found in this study. First, the sample size might need to be increased to show differences in MD. Second, HR was reduced by 11%, resulting in a proportional reduction in the MD value. Lastly, because this is a clinical study, values were only recorded for up to 20 min, and changes in CO and MD could occur later in anaesthetised dogs.

The use of these parameters (i.e., VTI and PV) allows for real-time assessment of the patient’s hemodynamic status and enables quick detection of changes [21,22,23,32,33,34]. This can be particularly valuable during anaesthesia in a clinical setting, as changes can occur rapidly, and more accurate yet invasive methods such as pulmonary artery thermodilution are not commonly available.

Pimobendan causes an increase in inotropy mediated by increased cAMP at the cardiomyocyte level and peripheral vasodilatation due to a reduction in PDE III activity. Both phenomena can increase systolic output, hence VTI and SV [10,11]. Based on the results of this study, the increase in VTI may have been primarily due to an increase in cardiac inotropism. Although systemic vascular resistance (SVR) was not assessed in this study, no changes in MAP were observed. This may indicate that pimobendan had little effect on blood vessels during the first 20 min.

Cardiac contractility can be assessed using invasive methods, such as placing an intraventricular pressure catheter. This catheter can measure the maximal rate of rise of left ventricular pressure (LV dP/dtmax). This parameter is widely used to assess contractility in several species [39,40,41]. The effects of pimobendan on inotropism appear less pronounced in anaesthetised dogs than in awake dogs [38]. In the present study, PV and MA were used to assess changes in inotropism: PV is the maximum velocity of blood in the aorta, and MA is the slope of the aortic velocity curve. These two parameters have previously been used to assess cardiac inotropism and have proven to be good indicators of a range of contractility situations in humans, mice, and dogs [39,40,41]. PV and MA reasonably correlate with LV dP/dtmax [42]. Studies in healthy dogs showed that using pimobendan caused a significant increase in LV dP/dtmax five and 10 min after administration [14]. The results of our study show that inotropism increases from the first minute after pimobendan administration and is maintained for at least the first 20 min. These results indicate a rapid increase in contractility in dogs with no cardiac disease under general anaesthetic. It is the first time that such immediate effects of pimobendan in dogs have been described. Additionally, pimobendan may also hold potential interest in senior animals with concurrent infectious or inflammatory diseases, as it is known that contractility, as well as systolic function, can be reduced in these states [6].

Systemic blood pressure and HR may be affected by using pimobendan in dogs. One study reported an increase in MAP and HR within 20 min of intravenous administration [16]. Another study showed an increase in HR 60 min after administration, with no change in MAP [15]. It should also be noted that when hemodynamic changes during anaesthesia and surgery are being assessed, MAP and HR could be affected by other factors, such as the cardiovascular effects of anaesthetic, analgesic, or sedative drugs or sympathetic responses to nociceptive stimuli. The dogs involved in the study underwent anaesthesia and elective surgeries. Consequently, medications commonly employed in routine clinical practice were utilized. Dexmedetomidine was administered to all animals as a pre-anaesthetic medication, known for its pronounced cardiovascular effects such as bradycardia and vasoconstriction. Additionally, other drugs such as methadone, alfaxalone, and isoflurane were employed, which can induce bradycardia, vasodilation, and reduced contractility of the heart. However, it is important to note that these medications were administered to both the placebo and pimobendan groups, indicating that their influence on the results should be minimal. Furthermore, during data collection for analysis, no changes in MAP or HR were observed.

This study has several limitations that should be considered when interpreting the results. First, the parameters for assessing contractility were measured in the descending aorta. The results could have been different if cardiac contractility had been measured by invasive methods at the intraventricular level. All haemodynamic parameters studied in this study (i.e., PV, MA, and VTI) are affected by cardiac contractility and preload and afterload [40,41,42]. Preload was not assessed in this study, but there were no signs of hypovolemia in any dogs on pre-anaesthetic examination and anamnesis. No other signs of preload dependence were detected during anaesthesia. Changes in pleural pressure during mechanical ventilation have essential effects on preload status and, thus, on CO. In the present study, neither pleural nor oesophageal pressure (as a subrogate) was recorded in the animals, so we cannot rule out differences between the two groups. Afterload was also not explicitly assessed by the SVR measured, but the absence of changes in blood pressure suggests that little change occurred at the vascular level. Finally, as this was a clinical study, it was only possible to record the hemodynamic effects of pimobendan during the first 20 min as most surgeries were elective procedures of short duration. Moreover, we did not employ a pulmonary artery catheter for hemodynamic monitoring as it is considered too invasive for this study. Furthermore, the different surgical procedures and locoregional anaesthesia techniques could introduce bias in the results. It is also important to note that cardiac surgeries were not included in the study, as in some cases, animals may already be receiving pimobendan prior to the surgery. Additional studies should evaluate these effects in more lengthy procedures and without surgical stimulation.

## 5. Conclusions

The use of IV pimobendan during general anaesthesia in senior dogs, after premedication with dexmedetomidine and methadone, increases systolic flow and contractility parameters, as measured using an oesophageal Doppler monitor. These changes occur from the first minute after administration. Both HR and BP remain unchanged. Based on the results of this study, pimobendan could be used to improve left ventricular function during general anaesthesia in senior dogs without cardiac pathology.

## Figures and Tables

**Table 1:** Demographic data of the studied groups. Age and weight are expressed as median [minimum, maximum]. ASA is shown as a frequency table.

	PLACEBO (N = 15)	PIMOBENDAN (N = 18)	Overall (N = 33)	*p*-Value
**Age**	9.00 [7.00, 12.0]	9.00 [7.00, 12.0]	9.00 [7.00, 12.0]	0.2
**Weight**	9.50 [5.00, 25.0]	11.5 [5.00, 25.0]	11.0 [5.00, 25.0]	0.8
**ASA**				
**1**	5 (33.3%)	2 (11.1%)	7 (21.2%)	0.12
**2**	10 (66.7%)	16 (88.9%)	26 (78.8%)	

**Table 2:** Descriptive statistics of the studied variables in PLACEBO and PIMOBENDAN groups over time. Data are expressed as median [range]. Heart rate (HR), mean arterial pressure (MAP), velocity-time integral (VTI), peak velocity (PV), mean acceleration (MA), minute distance (MD), end-tidal carbon dioxide tension (PE’CO_2_), end-tidal of isoflurane (FeISO). Values were measured one minute before pimobendan or placebo administration (T0), one min after pimobendan or placebo administration (T1), 10 min (T2) and 20 min (T3) after administration.

VARIABLE (Units)	Group	Time				*p*-Value
		T0	T1	T2	T3	
**HR (bpm)**	PLACEBO	74 [49, 138]	72 [48, 132]	72. [50, 133]	63 [51, 128]	*p* = 0.12
	PIMOBENDAN	86 [45, 102]	82 [43, 99]	78 [48, 110]	77 [48, 110]	
**MAP (mmHg)**	PLACEBO	86 [65, 138]	90 [64, 133]	88 [65, 141]	80 [59, 137]	*p* = 0.19
	PIMOBENDAN	83 [65, 156]	83 [60, 151]	80 [62, 143]	80 [74, 94]	
**VTI (cm)**	PLACEBO	10.6 [5.00, 15.9]	10.0 [4.60, 16.6]	9.80 [5.20, 14.7]	10.5 [6.50, 17.4]	*p* = 0.019
	PIMOBENDAN	10.9 [4.10, 18.8]	11.1 [4.90, 20.8]	12.4 [6.60, 21.1]	13.0 [10.4, 22.3]	
**PV (cm/s)**	PLACEBO	82 [47, 120]	83 [44, 123]	70 [61, 108]	80 [62, 103]	*p* < 0.0001
	PIMOBENDAN	88 [42, 141]	92 [48, 151]	101 [59, 156]	95 [83, 160]	
**MA (cm/s^2^)**	PLACEBO	9.7 [6.4, 18.5]	9.0 [6.7, 18.8]	9.9 [6.3, 15.6]	10.2 [7.0, 16.0]	*p* < 0.0001
	PIMOBENDAN	11.5 [6.3, 16.1]	12.0 [6.6, 19.0]	12.2 [8.3, 20.2]	12.6 [9.4, 17.0]	
**MD (cm)**	PLACEBO	774 [285, 1150]	792 [253, 1090]	778 [286, 1120]	831 [332, 1010]	*p* = 0.65
	PIMOBENDAN	823 [362, 1790]	785 [357, 2000]	969 [461, 1770]	1030 [499, 1870]	
**PE’CO_2_ (mmHg)**	PLACEBO	39 [35, 41]	39 [35, 44]	39 [36, 43]	40 [36, 42]	*p* = 0.23
	PIMOBENDAN	40 [35, 41]	40 [35, 42]	40 [35, 43]	40 [35, 41]	
**FeISO (%)**	PLACEBO	1.1 [1.0, 1.4]	1.1 [0.9, 1.4]	1.1 [0.9, 1.8]	1.1 [0.9, 1.4]	*p* = 0.08
	PIMOBENDAN	1.1 [0.7, 1.4]	1.0 [0.7, 1.4]	1.1 [0.7, 1.4]	1.1 [1.0, 1.2]	

## Data Availability

The data presented in this study are available on request from the corresponding author upon reasonable request.
